# Traumatic neuroma of the bile duct: A case report

**DOI:** 10.1002/ccr3.4619

**Published:** 2021-08-21

**Authors:** Salwa Nechi, Abdelwahab Nakhli, Wiem Ben Hamida, Amina Bani, Amal Khsiba, Asma Ben Mohamed, Emna Chelbi, Lamine Hamzaoui, Hassan Touinsi

**Affiliations:** ^1^ Department of Pathology Mohamed Tahar Maamouri Hospital Nabeul Tunisia; ^2^ Gastroenterology Department Mohamed Tahar Maamouri Hospital Nabeul Tunisia; ^3^ General surgery Department Mohamed Tahar Maamouri Hospital Nabeul Tunisia

**Keywords:** bile duct, cholecystectomy, jaundice, Traumatic neuroma

## Abstract

We report the case of a bile duct traumatic neuroma in a 76‐year‐old man who presented with obstructive jaundice one year after cholecystectomy. Despite the radiological examinations, the preoperative diagnosis was difficult. The patient underwent a biliary resection with choledoco‐duodenal anastomosis

## Graphical abstract

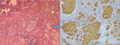

## 1 INTRODUCTION

A traumatic neuroma is a nonneoplastic lesion that develops in response to injury or surgery. It consists of a disorganized proliferation of a cutted nerve end. This lesion can develop following any surgery, especially amputation. Cholecystectomy has been rarely implicated in its pathogenesis^1^ with nonspecific clinical and radiological presentation that can mimic malignant tumors.

We present a case of a bile duct traumatic neuroma occurring after cholecystectomy. The clinical presentation was a biliary stenosis whose benign or malignant nature could not be confirmed preoperatively.

## 2 CASE REPORT

A 76‐year‐old man, who underwent a laparoscopic cholecystectomy one year ago, presented for jaundice without abdominal pain or fever. Physical examination revealed a jaundice. Vital signs were stable. The abdomen was soft with no evidence of organomegaly. Laboratory tests showed bilirubin: 20 mg/l (normal range <10 mg/l), alkaline phosphatase 524 U/L (1.9× ULN), and gamma‐GT 1001 U/L (18× ULN).

Abdominal ultrasound showed a dilation of intra‐ and extrahepatic bile ducts. The common bile duct measured 12mm, and the main bile duct was not dilated. The transition zone was the site of a small hypoechoic nodule measuring 5 mm.

**FIGURE 1:**
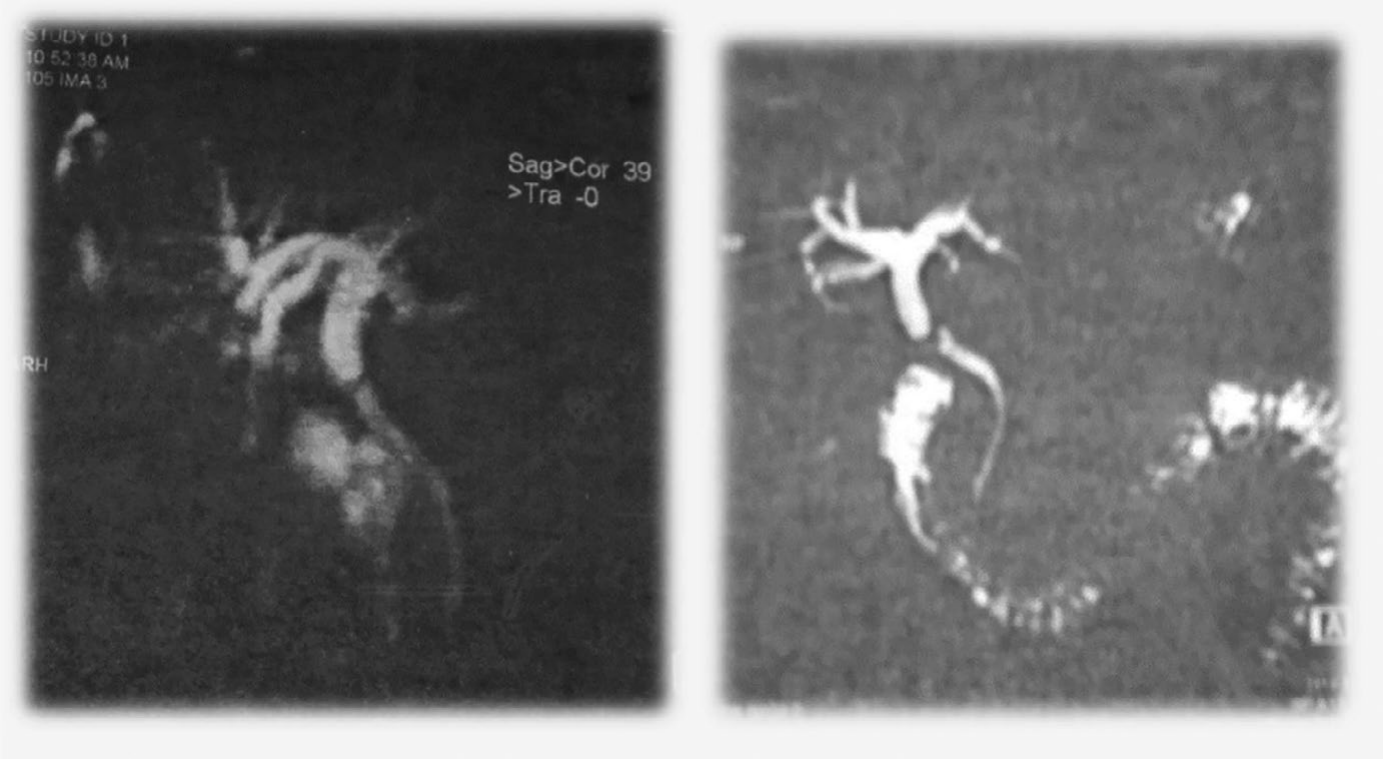
MRCP views showing a dilation of the common hepatic duct (9.6 mm) with a main bile duct measuring 5 mm.

A magnetic resonance cholangiopancreatography (MRCP) revealed (Figure 1) a dilation of the common hepatic duct (9.6 mm) with a main bile duct measuring 5 mm. The transition zone between the common hepatic duct and the main bile duct was the site of a small circumferential pseudomenbranous stenosis with no pathological enhancement.

The patient underwent a surgical resection of the main bile duct with a choledoco‐duodenal anastomosis.

The postoperative course was favorable. He was discharged from hospital on the fifth postoperative day.

**FIGURE 2:**
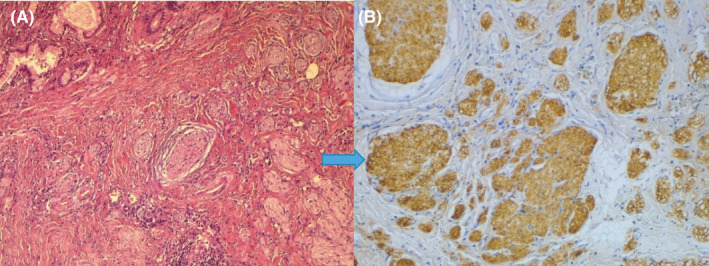
Histopathological examination. (A) Haphazard arrangement of nerves surrounding bile duct. (B) Nerve fascicles stain positively for PS100.

Pathology revealed traumatic neuroma of the bile duct with positive S100 stains and without any evidence of malignancy (Figure 2a and Figure 2b). At subsequent follow‐up, the patient reported no recurrence of biliary symptoms and liver tests were normal.

## 3 DISCUSSION

Traumatic neuromas are benign tumors that can develop after nervous system trauma.^1^, ^2^ They mostly occur in limbs and neck.^3^, ^4^ Traumatic neuromas in the biliary system are relatively rare.^5^ In a published study, up to 10% of cholecystectomized patients have a bile duct neuroma at autopsy.^2^ They can grow in the cystic duct stump after both laparoscopic and open cholecystectomy.^2^

The period between cholecystectomy and the onset of the neuromas varies from several months to 45 years.^2^, ^5^ The majority of patients remain asymptomatic.^2^

They may also present with a jaundice due to obstruction or with an intermittent right upper quadrant pain due to sympathetic nerves stimulation.^2^, ^5^

Imaging shows dilatation of the biliary ducts. MRCP can reveal the bile duct stenosis.^7^ The nerve tissue signal intensity is similar to the pancreatic head signal; thus, the diagnosis of biliary small traumatic neuromas can be difficult, especially if there is not enough fatty tissue around the bile duct.^7^

Endoscopic ultrasonography fine needle aspiration (EUS‐FNA) can be effective in the diagnosis of biliary stenosis (accuracy 68%–91%).^8^ Intraductal ultrasonography (IDUS) and peroral cholangioscopy (POCS) have also been reported to be effective in traumatic neuromas.^5^, ^9^ But further studies would be required to validate these techniques for traumatic neuromas diagnosis.^5^

Thus, despite all the modern imaging techniques, the certain preoperative diagnosis of traumatic neuroma is difficult.^1^, ^2^, ^5^, ^6^ Indeed, several differential diagnoses such postinflammatory fibrosis, retained stones, postoperative scar stricture, cholangiocarcinomas, metastatic lymph nodes, and neuroendocrine tumors must be excluded.^1^, ^2^, ^5^ Hence, surgical resection is the recommended approach for treatment and histological diagnosis^1^, ^2^, ^5^, ^6^

Neuroma formation affects nerves that are encased in Schwann cells. It has been suggested that it is caused by an increased levels of fibroblast growth factor and its receptor.^2^ Macroscopically, they are small white‐gray nodules seldom exceeding 5cm in diameter that develop at the end of the injured nerve. It is an exuberant but nonneoplastic proliferation of the nerve. Histologically, there are a disorganized proliferation of axons, schwann cells, and perineurial cells haphazardly embedded in a fibro‐collagenous background^2^, ^6^ with strong and diffuse immunostaining to S100 protein.

Considering that cholangiocarcinoma is one of the differential diagnoses, an aggressive resection is recommended: extrahepatic bile duct resection with negative margins and periportal lymphadenectomy.^2^

Some authors recommend using intraoperative pathologic examination to exclude a malignant tumor and thus avoiding excessive surgery.^5^

## 4 CONCLUSION

Traumatic neuroma should be considered in the differential diagnoses of patients with jaundice who have had a previous cholecystectomy. Surgical resection would be the recommended approach for diagnosis and cure given that cholangiocarcinoma is one of the differential diagnoses. Traumatic neuroma could also explain right upper quadrant pain in cholecystectomized patients.

## CONFLICTS OF INTEREST

None declared.

## AUTHOR CONTRIBUTIONS

Dr Salwa Nechi was involved in the definition of intellectual content and prepared the manuscript. Dr Abdelwaheb Nakhli designed the concept, was involved in the definition of intellectual content, in the literature search and preparation of the manuscript. Dr Wiem Ben Hamida gathered the information and assisted in the preparation of the manuscript. Dr Amina Bani extracted imaging data and assisted in the preparation of the manuscript. Dr Amal Khsiba assisted in the preparation of the manuscript. Dr Asma Ben Mohamed contributed to the critical review of the manuscript. Dr Emna Chelbi contributed to the critical review of the manuscript. Dr Lamine Hamzaoui was involved in the definition of intellectual content and contributed to the critical review of the manuscript. Dr Hassan Touinsi revised the manuscript and acted as guarantor for the research.

## ETHICAL STATEMENT

Written informed consent was obtained from the patient for publication of this case report and any accompanying images.

## Data Availability

Data are available as part of the article, and no additional source data are required.
